# Case report: Bullous pemphigoid associated with sintilimab therapy for pMMR/MSS colorectal cancer

**DOI:** 10.3389/fonc.2023.1124730

**Published:** 2023-03-14

**Authors:** Ting Wang, Qianwen Shao, Chunmei Xiao, Lianke Liu

**Affiliations:** Department of Oncology, The First Affiliated Hospital of Nanjing Medical University, Nanjing, China

**Keywords:** bullous pemphigoid, PD-1, fruquintinib, sintilimab, pMMR/MSS mCRC

## Abstract

Immunotherapy has become a very effective treatment for many cancers. It has a unique set of immune system-related adverse effects, collectively known as immune-related adverse events (irAEs). Skin toxicities are the most common irAEs, of which bullous pemphigoid, although rare, is potentially life-threatening and affects patients’ survival. In this article, we report the treatment of bullous pemphigoid caused by programmed cell death protein-1 (PD-1) in a case of proficient mismatch repair (pMMR)/microsatellite stable (MSS) colorectal cancer. No significant adverse effects were observed in the patient after methylprednisone was tapered to 4 mg twice a day. No new skin lesions occurred recently in the patient and the original skin lesions healed. In particular, the patient’s immunotherapy was not stopped and the best outcome was a partial remission of the disease, lasting for more than 8 months.

## Introduction

The immune checkpoint inhibitors (ICIs) play a vital role in the treatment of malignant solid tumors. Sintilimab is a humanized monoclonal antibody immunoglobulin G4 (IgG4) programmed cell death protein-1 (PD-1) antagonist that blocks the binding of PD-1 to programmed cell death ligand 1 and 2 (PD-L1 and PD-L2), relieving the immunosuppressive effect and activating the function of T cells, and has become widespread in clinical practice in China (1, 2). However, ICIs can cause cells to attack normal cells, causing a range of inflammatory diseases. ICIs may consume regulatory T cells (TREGs), resulting in the proliferation of antigen-specific B cells, and promote humoral responses at the level of lymphatic follicular germinal centers. The auto-reactive B lymphocytes produce antibodies against BP180 and BP230, leading to subepidermal disruption of the skin. All these implications eventually cause bullous pemphigoid. In bullous pemphigoid, the discontinuation of immunotherapy and the addition of steroids are recommended for all grades (3).

Here, we report a case of bullous pemphigoid caused by sintilimab in a proficient mismatch repair (pMMR)/microsatellite stable (MSS) colorectal cancer (CRC). We found that low-dose hormone therapy (methylprednisone, 4 mg twice a day) could control the skin lesions, ensuring the long-term use of sintilimab that brought patients more clinical benefits. Sintilimab and fruquintinib may be effective options for pMMR/MSS CRC.

## Case report

**Figure 1:**
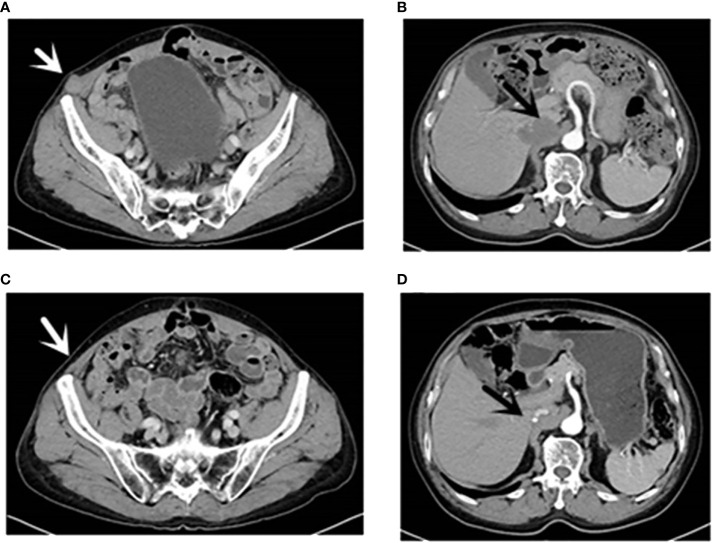
CT (16 August 2021): [**(A)**, white arrow] Right inferior abdominal wall metastases; [**(B)**, black arrow] hepatic caudate and inferior vena cava metastases and inferior vena cava invasion. CT (13 April 2022): [**(C)**, white arrow] Right inferior abdominal wall; [**(D)**, black arrow] the caudate lobe of liver and inferior vena cava. CT, computed tomography.

**Figure 2:**
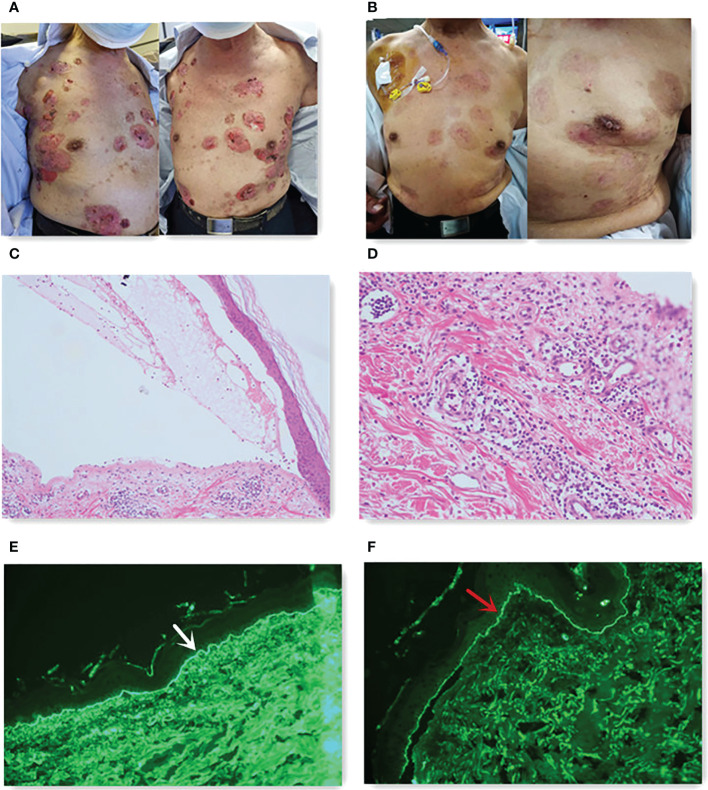
**(A)** Skin lesions before steroids. **(B)** Skin lesions after 10 months of steroids. **(C)** Subepidermal blister (hematoxylin and eosin staining, 40×). **(D)** Eosinophils existed in the blister (hematoxylin and eosin staining, 100×). DIF studies showed linear deposition of C3 [**(E)**, white arrow] and IgG [**(F)**, red arrow] along the basement membrane zone.

A 70-year-old man had a rectal mass by colonoscopy due to bloody stool and underwent radical resection (Dixon operation) in December 2017 (**Table 1**). The tumor size was 2.5 cm × 2.2 cm × 1.5 cm. The tumor invaded the fibrofatty tissue around the intestine, local vascular thrombus, and nerve. The stage was pT3N0M0 IIA. The patient only received capecitabine for 5 months. In November 2019, computed tomography (CT) revealed a mass in the right adrenal region (3.6 cm × 5.7 cm). In December 2019 (**Table 1**), CT-guided puncture biopsy of the adrenal mass was performed, and pathology indicated adenocarcinoma. Right retroperitoneal mass resection was performed in January 2020 (**Table 1**). The patient received oxaliplatin plus capecitabine for five cycles from March 2020 to August 2020. In January 2021, CT revealed a 2.5 cm × 1.7 cm nodule in the right lower abdominal wall, a filling defect in the inferior vena cava, and an invasion of the right diaphragmatic foot, indicating progressive disease (PD). Ultrasound-guided mass puncture in the right lower abdominal wall was taken in January 2021 (**Table 1**). The gene results of the mass tissue of the right lower abdominal wall indicated KRAS p.G12D (72.9%), MLH1 (−), MSH2 (−), MSH6 (−), PMS2 (−), tumor mutation burden-low (TMB-L) 8.64 Muts/Mb, and MSS. The immunohistochemistry (IHC) of PD-L1 was 60% tumor proportion score (TPS) and 60 combined positive score (CPS). Six cycles of irinotecan, S-1, and bevacizumab were performed from March 2021 to July 2021. CT was evaluated in April 2021 and June 2021, suggesting stability of disease (SD). In August 2021, CT revealed a metastatic tumor in the caudate lobe and inferior vena cava area of the liver, showing PD. From August 2021, he received fruquintinib at a dose of 5 mg once a day for 21 days, every 28 days, and a fixed dose of sintilimab (200 mg) every 3 weeks. In April 2022, he achieved partial remission (PR) after 10 cycles (**Figure 1**). The patient developed erythema and pimples accompanied by pruritus on the chest, abdomen, and right leg since January 2022 after 5 months of use of sintilimab plus fruquintinib. The lesions subsequently aggravated, forming strained blisters filled with serous fluid (**Figure 2A**) in February 2022. He took topical corticosteroids for a few days but the lesions worsened. He came to the dermatology clinic for a skin biopsy. Hematoxylin and eosin (HE) staining showed subepidermal blisters, necrosis of the upper epidermal wall, and serous exudation (**Figure 2C**). A large number of eosinophils were present in the blister, while lymphocytes, monocytes, and eosinophils were infiltrated around the vessels in the lower dermal vasodilation (**Figure 2D**). Direct immunofluorescence (DIF) revealed the linear deposition of C3 (**Figure 2E**) and IgG (**Figure 2F**) along the basement membrane zone. IgG antibodies were positive for BP180 antigen with 120.0 U/ml titer by enzyme-linked immunosorbent assay (ELISA). BP230 detection was negative. His lesions were classified as grade 2 because of a body surface area of 19% according to Common Terminology Criteria for Adverse Events (CTCAE) version 5.0. He had mild hypertension without medication. He had a partial resection of the thyroid gland. His father suffered from gastric cancer.

**Table 1:** Immunohistochemical results of four tumor tissues from the patient.

Tumor tissue	Immunohistochemistry (IHC)
Rectal mass (December 2017)	MLH1 (+), MSH2 (+), MSH6 (−), PMS2 (+), EGFR (+), HER2 (+), local Dog - 1 (−)
CT-guided puncture biopsy of the adrenal mass (December 2019)	CK7 (−), CK20 (+), CDX-2 (−), Ki67 (50–90%+), Villin (+), TTF-1(−)
Right retroperitoneal mass (January 2020)	CK7 (−), CK19 (+), CK20 (+), Villin (+), CDX-2 (focus +), CDH17 (focus +), SATB2 (−), CD56 (−), CgA (−), Syn (−), AMACR/p504s (−), AR (−), GATA3 (−), PSA (−)
Ultrasound-guided mass puncture in the right lower abdominal wall (January 2021)	CK7 (−), CK20 (+), CDH17 (+), CDX-2 (+), SATB2 (focus +), Villin (+), Ki67 (45% +)

**Figure 3:**
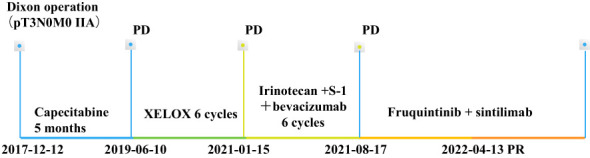
Timeline of the treatment processes. XELOX, oxaliplatin + capecitabine. PD, progressive disease, PR, partial response.

We promptly gave the patient oral methylprednisolone 12 mg twice daily. The patient extended the interval of sintilimab for 5 weeks due to the pain from blisters and sores; however, he took fruquintinib regularly. He made a strong request for sintilimab and signed an informed consent that anti-PD-1 might worsen his bullous pemphigoid. Methylprednisolone was tapered to 4 mg orally twice daily without lesion recurrence (**Figure 2B**). However, the blisters immediately appeared as soon as the patient tried to stop or decrease the dose of methylprednisolone. We recommended rituximab for the patient, but he refused because of financial reasons. The patient saw the dermatologist monthly. We performed immunotherapy with the assistance of the dermatologist. At the time of writing this report, the patient’s rectal cancer reached partial response (**Figure 3**) with a progression-free survival (PFS) of 8 months (we expected a longer PFS).

## Discussion

The incidence of CRC is the third highest among all types of cancer worldwide, and prognostic outcomes have remained to be poor. The role of immunotherapy in metastatic CRC (mCRC) is still under investigation. Only 5% of mCRC cases with defect mismatch repair (dMMR)/microsatellite altitude instability (MSI-H) may benefit from ICI treatment. However, most patients (95% of mCRC) with pMMR/MSS tumors do not respond to current immunotherapy. The effects of anti-PD-1/PD-L1 antibody combined with vascular endothelial growth factor receptor (VEGFR) tyrosine kinase inhibitors (TKIs) are attractive research directions for mCRC. VEGF promotes cancer immune resistance through a complex matrix. Studies have shown that possible mechanisms include inhibiting CD8^+^ T-cell activation, increasing checkpoint molecular expression, inhibiting dendritic cell maturation, decreasing Major Histocompatibility Complex (MHC) expression, and recruiting myeloid suppressor cells. Inhibition of VEGF can reverse cancer immune resistance (4). Fruquintinib is a small anti-VEGFR TKI that targets VEGFR-1, -2, and -3, and has been approved by the National Medical Products Administration (NMPA) of China in 2018 for third-line or later treatment in patients with mCRC based on the Fruquintinib Efficacy and Safety in 3+ Line Colorectal Cancer Patients (FRESCO) (5). The fruquintinib and sintilimab combination was superior to fruquintinib monotherapy in the treatment of mCRC (ORR: 15.4% vs. 4.9%) according to an American Society of Clinical Oncology (ASCO) study published in 2020 (6). The 2021 ASCO Meeting Abstract revealed that the objective response rate (ORR) was 27.3% and the PFS was 6.9 months in the fruquintinib 5-mg group, though it was a phase Ib/II study of the fruquintinib and sintilimab combination for advanced CRC (7). Caiyun Nie suggested that patients with MSS mCRC without liver metastasis responded well to the fruquintinib plus sintilimab regimen (ORR: 21.4%) (8). We recommend the combination of fruquitinib and sintilimab for the patient based on recent clinical trials, and our center has received several successful treatment cases.

Bullous pemphigoid has a profound impact on anti-PD-1 therapy. How to identify bullous pemphigoid early is particularly important to improve the prognosis of patients. To identify bullous pemphigoid, a skin biopsy is recommended, which should be obtained from the non-bullae portion surrounding the lesion within 1–2 cm. Histopathologic features include the distribution of eosinophils and/or neutrophils at the subepithelial cleft. DIF shows linear deposition of IgG and/or C3 along the dermo-epidermal junction, and sometimes IgA and IgE have similar shapes. The antibodies against the hemidesmosomal protein BP180 and sometimes BP230 antigens through ELISA are also characteristics (9). In most cases, the diagnosis of bullous pemphigoid relies on clinical characteristics of skin lesions, positive DIF (linear deposits of IgG and/or C3 along the dermo-epidermal junction, sometimes IgA and IgE), and BP180 and/or BP230 by ELISA or indirect immunofluorescence microscopy (IIF).

The treatment of bullous pemphigoid is correlated with the severity of the disease ranging from grade 1 to the most severe case (grade 4) according to the CTCAEs (10). Cessation of immunotherapy is recommended for all grades of bullous pemphigoid; however, for grades 2–3, permanent discontinuation of immunotherapy is recommended. For grade 1, highly effective topical steroids are recommended, while for grades 2–3, systemic corticosteroids are required, prednisone 0.5–1 mg/kg/day is recommended, and, for those whose disease is not under control for 1–3 weeks, prednisone dose is increased up to 2 mg/kg/day (11, 12). If steroid contraindications or resistance is present, immune-suppressive therapies, including methotrexate (13), azathioprine (11), mycophenolate mofetil (14), or mycophenolate acid (12), may be recommended. Monoclonal antibodies such as rituximab (anti-CD20) (15, 16), omalizumab (anti-IgE) (17) and dupilumab (anti-IL-4Rα) (18), and immunoglobulin and immunosorbent (19, 20) are also treatment options.

Whether systemic corticosteroids attenuate the anti-tumor immune response remains to be determined. Among patients with melanoma who had ipilimumab-induced hypophysitis, those who received higher doses of corticosteroids had reduced survival (21), suggesting that low-dose steroids (a maximum average daily dose of 7.5 mg prednisone or equivalent) are more beneficial for overall survival (OS) (22). In our case, methylprednisone (4 mg orally twice daily) therapy ensured the continuation of immunotherapy without obvious side effects, creating an enduring state of immune activation that contributed to the maintenance of tumor response, which is consistent with the fact that almost all the current anti-PD-1/PD-L1 clinical studies do not exceed 10 mg/day of prednisone or other equivalent doses of hormones.

We believe that fruquintinib is not the agent that triggers bullous pemphigoid. In the FRESCO trial, hypertension, hand–foot skin reaction, and proteinuria were the most common treatment-related adverse events occurring in patients with mCRC treated with fruquintinib (23). No bullous pemphigoid has been reported in patients treated with fruquintinib. The fact that skin lesions of the patient improved with steroid therapy and relapsed when steroid therapy stopped suggests that bullous pemphigoid might be an adverse reaction to sintilimab.

In summary, we first bravely tried to use methylprednisone (4 mg orally twice daily) to control bullous pemphigoid, and the patient’s long-term combination of sintilimab and fruquintinib resulted in a longer period of PR; this differs from the generally accepted recommendation to permanently discontinue immunotherapy. We have provided an effective case for sintilimab and fruquintinib in the treatment of pMMR/MSS CRC. However, the small number of cases and the lack of skin biopsies from patients in remission prevented us from further studying bullous pemphigoid.

## Data availability statement

The raw data supporting the conclusions of this article will be made available by the authors. Further inquiries can be directed to the corresponding authors.

## Ethics statement

Written informed consent was obtained from the individual(s) for the publication of any potentially identifiable images or data included in this article.

## Author contributions

TW drafted the manuscript and provided figures, CX provided figures, and LL revised the manuscript critically. QS managed the clinical treatment of the patient. All authors contributed to the article and approved the submitted version.
